# Hypertrophy of the Follicular Glands at the Base of the Tongue

**Published:** 1894-10

**Authors:** Horace Clark

**Affiliations:** 21 West North Street; Buffalo


### HYPERTROPHY OF THE FOLLICULAR GLANDS AT THE BASE OF THE TONGUE.

By HORACE CLARK, M. D., of Buffalo.

Clinical study of disease of the glands at the base of the tongue is of comparatively recent date, although attention was called to the subject as early as 1877. The general observer has not yet given it the attention which its importance merits. A moderate degree of hypertrophy of these glands being commonly found without attendant symptoms, a more pronounced enlargement escapes attention, and search is apt to be made elsewhere for an explanation of the symptoms incident thereto. The importance of accurate examination of this region cannot be overestimated when the clinical history is such as will be described later on in this paper.

The presence of follicular glands at the base of the tongue is normal and constant in adults. Their aggregation into the so-called *lingual tonsil* is analogous to that between the pillars of the fauces forming the *faucial*, and in the vault of the pharynx the *pharyngeal* tonsil. At the base of the tongue, however, the glands are not aggregated into large thick masses, but are seen as little rounded cushion-like elevations, irregularly arranged, with smooth areas interspersed. They are most numerous in the neighborhood of the papilla circumvalata, where they seem to form true papilla, extending backwards to the glosso-epiglottic sinuses and to the right and left toward the faucial tonsils. In the middle of each gland is a slight hollowing out, which is the orifice of an excretory sinus, leading to a pouch-like cavity which is lined by a prolongation of the mucous membrane from the orifice. Underneath this lie the lymph bodies embedded in a fine reticulum, the whole mucous gland being enclosed in a fibrous capsule. There are from thirty-two to one hundred and four of them. Their average size amounts to from one to five mm.

Hypertrophy of the follicular glands of the tongue is due, in general, to the same causes as that of the same sort of tissue in other parts of the upper respiratory tract. The fact that there is a continuity of lymphatic tissue, in the form of a ring, between the lingual, faucial and pharyngeal tonsils, would suggest a ready means of communication of disease from one to the other. That there may be a connection between this disease and such acute diseases as scarlet fever, measles, diphtheria and typhoid fever, is supported more from the results of *post mortem* examination than from clinical history. That these glands may be attacked by tuberculosis and become enormously enlarged, has been observed. The disease is one of adult life, or, at any rate, its symptoms do not manifest themselves until then. The youngest recorded case is sixteen years, the oldest fifty-seven years. It occurs more frequently in females. This may be due to the more pronounced nervous temperament among women. It is not frequent among the laboring classes. This is probably due to lack of accurate observation on the part of such patients, or, perhaps, to lack of careful examination on the part of the surgeon in the hurry of a public clinic. The course of the disease is that of a chronic inflammation. Instances of acute exacerbation are not commonly seen. The writer is strongly of the opinion that the location of these glands, predisposing them to irritation from food and drink, has some etiological value as to their hypertrophy. This observation is made from a number of clinical histories.

The symptoms occasioned by this condition depend almost wholly upon contact between the glands and the epiglottis, and the intensity of the symptoms is proportional to the extent of contact. The latter must be a variable quantity, because of the varying convexity of the tongue and the form and position of the epiglottis. The convexity may be very pronounced antero-posteriorly or transversely, or, the parts at the sides may be more convex than those in the middle. Thus there may be a very small amount of glandular hypertrophy, which, by reason of its situation, may call forth pronounced symptoms. Again, the epiglottis may be so far removed from contact as to produce no symptoms at all even in the presence of considerable hypertrophy.

The most characteristic, if not most frequent, symptom thus produced is that of a foreign body in the throat. The patient's description of this is very variable. He will often try to put his finger on a " lump " whose presence he is almost constantly aware of, causing tickling and coughing, but which he cannot locate exactly. Or, he says there is a place in his throat which feels as if a small piece of a stick were lodged there. A circumscribed roughened area is often mentioned. Whatever the description, a tickling and irritation is nearly always noted, and an effort to get rid of the offending material by coughing or hawking. The more this effort is persisted in the more ineffectual it becomes. In markedly developed cases, vomiting may sometimes supervene. I have made one such observation in the case of an elderly man, whose symptoms were especially aggravated by food and drink. During or after meals his throat would become so irritable that violent attacks of coughing would be set up, ending frequently in vomiting. Increased annoyance after eating is not infrequently complained of. The explanation of this may be found in a hyperemic condition of the glands, caused by hot or highly seasoned food. Coughing is not attended with much expectoration in uncomplicated cases.

*A sense of pressure* in the throat is a symptom very commonly given. This is most noticeable after long-continued singing or speaking, and may result in what is described as "hollow-swallowing." This sensation is precisely the same as that when a particle of food remains upon the base of the tongue after food has been swallowed, but not completely. Or, after having falsely swallowed, the sensation remains as if something had stuck in the throat.

*Tiring and impaired clearness of the voice*, notably complained of by singers, is probably due in part to mechanical interference with the free movement of the epiglottis, or to a sense of stiffening of the base of the tongue, impeding the vocal passage, and making a "throaty" tone. Or, the explanation may be found in the constant rubbing between the epiglottis and these glands. The nerves of the epiglottis are irritated and the voice is reflexly affected.

Huskiness of the voice, giving rise to a desire to *clear the throat*, may be caused by over-abundant secretion upon the cords. This may be simply a coincidence due to some other coëxisting lesion, producing slight inflammation of the cords. In a former paper[1] I pointed out how this occurs, and that the hypersecretion attendant upon adenoids, for example, is swallowed and does not pass on into the larynx. In the case of hypertrophied lingual glands, however, I do not see why the secretion, consequent upon friction, which collects in the glosso-epiglottic sinuses, should not

1. *Buffalo Medical and Surgical Journal*, May, 1893.

mechanically find its way into the larynx; or, similarly, the secretion of the glands themselves when they occupy these sinuses.

The following symptoms are also mentioned by several clinical observers: slight pain in the throat accompanied by a burning sensation; a spreading of the sense of pressure toward the ears on swallowing, or along the esophagus, between the shoulders, to the entrance to the stomach, or into the larynx and trachea. The symptoms may be more annoying when there is cold in the head, constipation, or from hard manual labor, as lifting, etc. This is explained by increased blood pressure under these conditions. Finally, it should be borne in mind that the symptoms are influenced by the patient's general health, and that they are especially aggravated by anything tending to nervousness.

When the given subjective symptoms can be reasonably supposed to be traceable to hypertrophied glands at the base of the tongue, the laryngoscopic picture will be somewhat as follows: the surface of the tongue will be found more or less covered with excrescences, of rather soft consistency, varying in size from a small lead shot to a cherry pit, although they may be considerably larger. Some of these are isolated, or arranged in rows or irregular forms. Neighboring glands or groups of glands may have run together, the boundary between them being lost. Thus, here and there, a conglomerate, frequently cone-shaped mass, of irregular outline, is formed, overreaching the free border of the epiglottis and extending into the glosso-epiglottic sinuses. Cases are recorded in which the epiglottis was fairly imprisoned. The cords show slight inflammation.

In making the diagnosis, the point to be determined by examination is neither the number of these enlarged glands nor the size of the enlargement, but whether they are so situated as to come in contact with the epiglottis. As an aid to diagnosis I make a practice of touching with the probe both the glands and the epiglottis at the points of contact. Thus it often happens that the same sensation which the patient has complained of will be brought out. It has also been suggested to cocainize the part and observe whether the irritation is thus allayed.

It must be admitted that the above described relations may pertain and yet there need be no corresponding symptoms. The most that can be said is that when there are such symptoms and such a condition, the probability is that the trouble lies here, and is, therefore, to be attacked as the salient point, although there may be lesions elsewhere which might give rise to a very similar set of symptoms.

In the treatment of this disease, results are claimed from repeated applications of a preparation of iodine, iodide of potassium and glycerine, as well as from the various caustic acids, London paste, etc. These methods are to be considered only when operation for the immediate removal of the tissue is refused. Neither is the galvano-cautery alone advisable. This, too, may require repeated use, and the patient suffers considerable discomfort between times. Furthermore, it is difficult to avoid touching the epiglottis with the cautery. The cold snare is advocated as the most efficient instrument. A curved canula should be used. A small guillotine has been devised, worked like a tonsillotome with the "Schroetter" handle. It is difficult to make the blade catch. Cutting instruments, such as curved scissors, are objected to because of possible hemorrhage. I do not think they should be discarded for this reason if their use is preferred. If hemorrhage is to occur at all it must come from enlarged veins, which, indeed, may cause a varix in this region. These can be seen sufficiently well to remove danger from them before-hand by their destruction with the galvano-cautery. The looped lanyngeal curette has given me by far the smoothest resulting surface. The tissue is soft and is readily scraped away in small lumps. Any remaining unevennesses may be now removed by the galvano-cautery with very little danger to the epiglottis, because sufficient free space has been obtained between it and the base of the tongue. The soreness upon swallowing after operation is not serious and does not seem to be increased by this searing. A thin slough is formed which usually separates within three or four days. The wound should be cleansed for a few days subsequent to operation. At the same time, applications of the *etherial* tincture of the chloride of iron will be found to have considerable value in relieving the soreness.

21 West North Street.

---

Salicylate of sodium is recommended as little less than a specific in acute cases of tonsilitis. It should be given as early in the attack as possible, and in sufficient doses to cause ringing in the ears. Fifteen grains every three hours will usually cause this effect, when the dose may be diminished to ten, and then to five grains, at the same intervals. It should be continued for a day or two after disappearance of the fever.—*N. C. Medical Journal.*

